# Chemotherapy resistance and stromal targets in breast cancer treatment: a review

**DOI:** 10.1007/s11033-020-05853-1

**Published:** 2020-10-01

**Authors:** Y. M. van der Spek, J. R. Kroep, R. A. E. M. Tollenaar, Wilma E. Mesker

**Affiliations:** 1 Department of Surgery, Leiden University Medical Center, Albinusdreef 2, 2333 ZA Leiden, The Netherlands; 2 Department of Medical Oncology, Leiden University Medical Center, Leiden, The Netherlands

**Keywords:** Breast cancer, Tumor–stroma, Chemotherapy resistance, Stromal targets, Review

## Abstract

Therapy resistance is a known problem in breast cancer and is associated with a variety of mechanisms. The role of the tumor microenvironment in cancer development and resistance mechanisms is becoming increasingly understood. Tumor–stroma is the main component of the tumor microenvironment. Stromal cells like cancer-associated fibroblasts (CAFs) are believed to contribute to chemotherapy resistance via the production of several secreted factors like cytokines and chemokines. CAFs are found to influence disease progression; patients with primary tumors with a high amount of tumor–stroma have a significantly worse outcome. Therefore the role of CAFs resistance mechanisms makes them a promising target in anti-cancer therapy. An overview of recent advances in strategies to target breast cancer stroma is given and the current literature regarding these stromal targets is discussed. CAF-specific proteins as well as secreted molecules involved in tumor–stroma interactions provide possibilities for stroma-specific therapy. The development of stroma-specific therapy is still in its infancy and the available literature is limited. Within the scope of personalized treatment, biomarkers based on the tumor–stroma have future potential for the improvement of treatment via image-guided surgery (IGS) and PET scanning.

## Introduction

Breast cancer is the most commonly occurring type of cancer in women worldwide and is one of the greatest causes of female death [1]. The disease is heterogeneous and various treatment options are applied in clinical practice as local treatments, including surgery and radiotherapy, as well as chemotherapy. More specific therapies include hormone, targeted and immunotherapy. Still, therapy failure and disease recurrence due to drug resistance remain common in all breast cancer types [2].

**Fig. 1:**
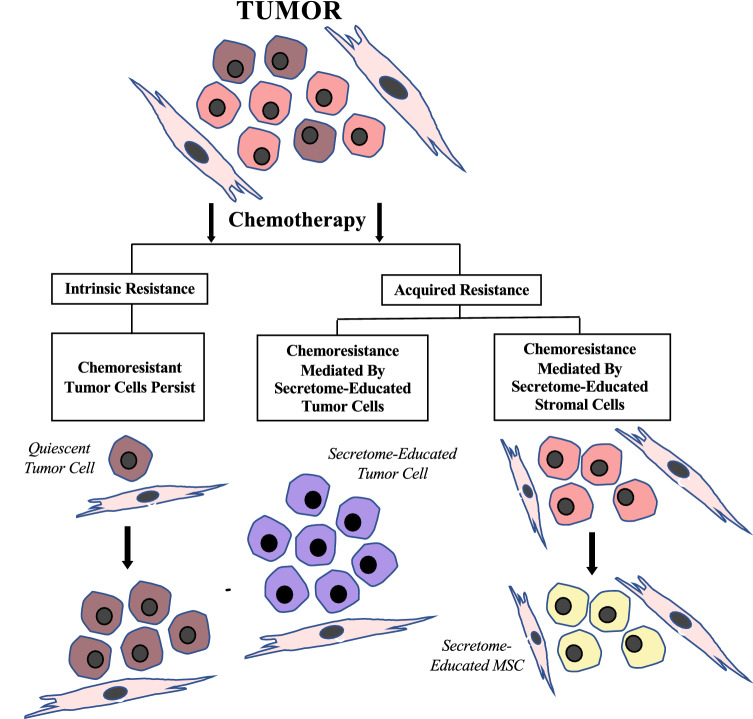
Schematic presentation of chemotherapy resistance mechanisms

Chemotherapy resistance can be an intrinsic and inherent feature of tumors. Resistance mechanisms of tumors were previously associated with tumor cell alterations, like altered membrane drug transport, altered DNA repair and altered apoptosis mechanisms [2, 3]. However, acquired resistance might also occur despite an initial good response to chemotherapy. These different resistance mechanisms are presented in Fig. 1. The role of tumor micro-environment (TME) in acquired chemo-resistance is increasingly understood. A variety of cells is found in the tumor micro-environment, including adipocytes, bone-marrow derived stem cells and several stromal cell types. These cells are embedded in the extracellular matrix (ECM) and receive blood by a vascular network. The TME contributes to the progression, metastasis and drug resistance of breast cancer. Contrariwise, tumor cells influence the phenotype of their TME, leading to complementary interactions between tumor cells and the TME including the tumor–stroma [4].

**Fig. 2:**
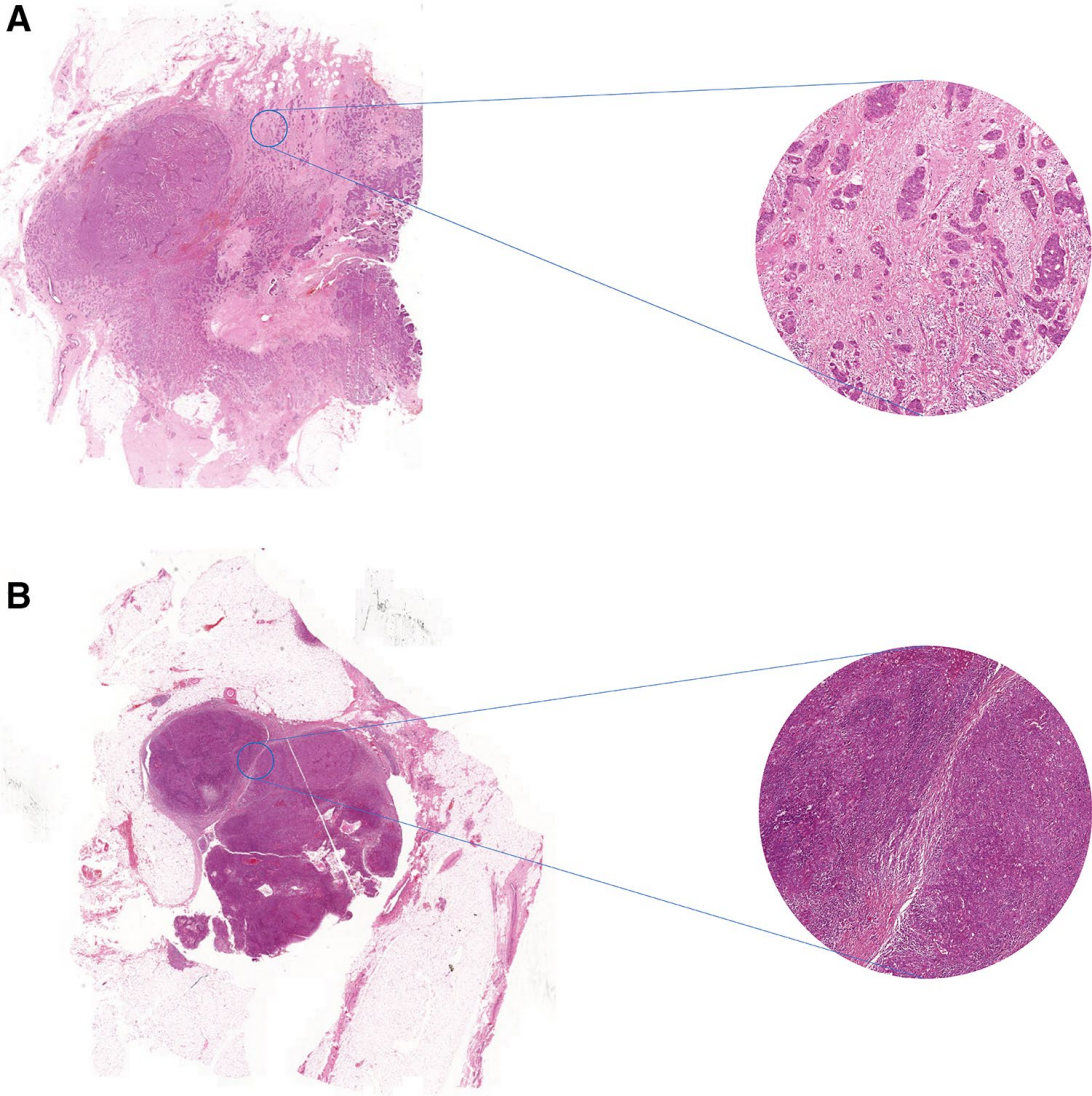
Microscopic images of breast tumor sections stained with hematoxylin and eosin. **A** Stroma-high and **B** stroma-low

The ratio between tumor cells and stromal tissue has been proven to quantitatively reflect the stromal processes contributing to tumor progression, invasion and metastasis [5]. Research has been conducted studying the prognostic value of the tumor–stroma ratio (TSR) in addition to standard clinical markers used in practice [6–11]. TNM staging is currently the gold standard [12]. Kramer et al. discussed five studies showing a significant correlation between poor prognosis and a high stromal content in breast cancer patients [13]. Assessment of the TSR of the primary tumor combined with tumor–stroma positive lymph nodes provides additional prognostic information [5]. TSR scoring involves an easy microscopic technique, quantifying the amount of stroma present in a tumor tissue slide [13, 14]. Microscopic images of stroma-high and stroma-low breast tumor sections are shown in Fig. 2.

Among others, stromal cells like cancer associated fibroblasts (CAFs) contribute to chemotherapy resistance. Induction of the cell-cycle arrest, epithelial–mesenchymal transition or cancer cell proliferation are examples of chemo-resistance mechanisms. Direct interaction between tumor cells and the TME is involved in this process, especially through gap junctions, interacting proteins, immune cells and receptors [15]. Also, indirect communication occurs via the secretion of several components, including cytokines and exosomes [16]. These insights into the complex mechanisms of chemoresistance and the role of tumor–stroma provide possibilities for new targeted strategies in breast cancer therapy.

In this review, the influence of tumor–stroma on tumor development, invasion and metastasis and on therapy resistance is described. The current literature investigating stromal targets in personalized breast cancer treatment is discussed and future possibilities and suggestions for further research are considered.

## Cancer-associated fibroblasts

Cancer-associated fibroblasts (CAFs) are a major component of the cancer stroma. Cancer-associated fibroblasts are derived from different origins. Among others, vascular smooth muscle cells, pericytes, adipose-derived mesenchymal stem cells and bone marrow-derived mesenchymal stem cells have been recognized as CAF progenitors [17]. These CAFs with different progenitors express distinct markers, like α-smooth muscle actin or fibroblast activator protein.

Fibroblasts in normal tissue are mostly involved in structural processes, as they secrete ECM proteins like fibronectin and collagen. Under normal conditions fibroblasts are in an inactivated state, but they can be activated by several processes like inflammation and injury when tissue remodeling is needed. Activated fibroblasts are abundant in tumors; in breast tumors this accounts for 80% of the total amount of CAFs [18]. Unlike normal fibroblasts, CAFs remain in this activated state. Genomic instability has been proposed as the possible cause of this constant activation and altered phenotype. Conflicting results have been found on this hypothesis, but these seem to be due to varying methods of tissue specimen processing. Determining the possible genomic alterations and instabilities of CAFs provides possibilities for CAF identification and might even be integrated in therapy response and resistance [19].

## Tumor–stroma interactions

Activated CAFs promote tumor proliferation, invasion and metastasis and induce angiogenesis via the production and secretion of several factors, including cytokines, growth factors and ECM components. These cytokines include vascular endothelial growth factor (VEGF), stromal cell-derived factor (SDF1) and transforming growth factor β (TGF-β) [20]. Moreover, secretion of ECM-degrading proteases is observed, contributing to the increased ECM-remodeling. Literature has also shown that CAFs are involved in chemoresistance of breast tumors. One of the mechanisms by which CAFs contribute to chemoresistance is the production of IL-6 and IL-8 that leads to the support of cancer stem cells, CSCs [21]. Furthermore, the chemokine CCL2 in breast cancer regulates the recruitment of macrophages. Moreover, CAFs can induce the epithelial–mesenchymal transition in tamoxifen-resistant breast cancer cells or induce resistance via the production of hyaluronan [22, 23]. These and other crucial functions of CAFs in disease progression and the development of chemoresistance make them a promising target in anti-cancer therapy. Besides this, eradication of CAFs could reduce the physical barrier and improve drug delivery to tumor cells.

## Stroma-specific therapy

### Stromal targets

Several strategies have been proposed to target breast cancer CAFs to improve anti-tumor therapy. Table 1 provides an overview of these stromal targets. The fibroblast activation protein alfa (FAPα) is one of the targets that is widely studied. This protein is selectively expressed by CAFs and is undetectable in normal stroma [24]. One strategy to target FAPα is via immunization. Vaccination with FAP-positive stromal cells inhibited allograft tumor growth, induced apoptosis and decreased collagen and CD31 expression in the TME of a murine breast cancer model [25]. DNA-vaccines expressing FAPα stimulate FAPα-specific cytotoxic T-cells that kill CAFs. This led to reduced tumor progression in a murine 4T1 breast cancer model and a decrease in the expression of collagen I and other stromal factors that promote tumor growth [26, 27]. However, no papers studying this specific targeting in human cancer models have been conducted.

**Table 1:** Overview of targets for stroma-specific therapy

Stroma-specific therapy	Author
*Stromal targets*	
Fibroblast activation protein alfa (FAPα) targeting via immunization	Meng et al. [25], Xia et al. [26], Geng et al. [27]
Nanomedicine	
Photodynamic therapy	Truffi et al. [24]
Nanoparticles combined with FAP-specific antibodies	Zhen et al. [28]
Nanoparticles for drug delivery	Ji et al. [29], Hu et al. [30], Zhu et al. [31]
*Targeting tumor–stroma interactions*	
GW4064 (farnesoid X receptor (FXR) agonist)	Giordano et al. [34]
Pirfenidone (PDF) combined with doxorubicin	Takai et al. [35], Polydorou et al. [36]
SMO-inhibitors (vismodegib, sonidegib)	Cazet et al. [39], Ruiz-Borrego et al. [40]
Targeting of amphiregulin	Xu et al. [41]
E5 (CXCL12 antagonistic peptide)	Guo et al. [44]
WRG-28 (small molecule inhibitor targeting DDR2)	Grither and Longmore [46]

Nanomedicine presents another promising approach to target CAFs. Several strategies in nanomedicine have been investigated, the first being photodynamic therapy [24]. Zhen et al. investigated the use of nanoparticle-based photoimmunotherapy to target CAFs. They combined ferritin nanoparticle protein cases and FAP-specific antibodies to successfully eliminate CAFs. Furthermore, ECM deposition and secretion of the chemokine CXCL12 diminished, facilitating the infiltration of T-cells. However, Zhen et al. did not specifically test this technique in breast cancer cells [28]. Nanoparticles might also be used for the delivery of cytotoxic drugs to cancer cells. Among others, the use of cleavable amphiphilic peptides (CAP) responsive to FAPα was investigated by Hu et al. [29]. Drug-loaded CAP-polymers were cleaved upon binding by FAPα, leading to the release of chemotherapeutics. This mechanism was shown to be effective in the treatment of prostate, breast and pancreatic tumor models. Furthermore, Hu et al. showed promising results of long filaments of peptide derivative nanofiber entrapping losartan in aggressive TNBC [30]. Zhu et al. subsequently administered glycolipid-based polymeric micelles (GLPM) encapsulating telmisartan, an angiotensin II receptor inhibitor, and doxorubicin. This resulted in decreased CAF activity and CAF-derived stroma. Activation of the apoptosis-related peroxisome proliferator-activated receptor-gamma (PPAR-γ) pathway induced a synergistic effect on breast tumor cells [31]. The fourth strategy aims at regulating the CAF function [24]. However, studies exploring this strategy have not been specifically tested for breast cancer and will therefore not be discussed in this review.

### Targeting tumor–stroma interactions

Stromal cells in breast cancer execute most of their effects on tumor cells via cytokines, growth factors and other secretable molecules [19]. Several studies have been conducted targeting these tumor–stroma interactions, that have been shown to be of importance for tumor growth and therapy resistance. A first potential target in tumor–stroma interactions is the adipokine leptin. Leptin and the leptin receptor ObR are overexpressed in breast cancer [32]. Leptin produced by stromal cancer-associated fibroblasts promotes the invasion, migration, proliferation and mesenchymal transition of breast cancer cells [33]. The farnesoid X receptor (FXR) has complex and contradictory functions in breast cancer regulation, but it has been shown that activation of this receptor inhibits leptin-induced breast cancer progression and motility. The FXR agonist GW4064 induced a decrease in the activation of the leptin signaling pathway and reversed the CAF-induced effects on tumor progression and motility. Furthermore, the autocrine amplification loop mediated by leptin and its receptor was reduced after administration of GW4064. This effect was shown both in vitro and in vivo in xenograft models [34].

Also pirfenidone (PDF), an anti-fibrotic compound, inhibits collagen production and tumor growth in 2D and 3D triple-negative breast cancer (TNBC) mouse models. TNBC is an aggressive breast cancer subgroup and treatment of this type is complicated, thus the clinical need for targeted therapies is high. The inhibitory effect of PDF is mostly regulated via TGF-β signaling pathways, suggesting an effect of PDF on tumor progression via tumor–stromal interactions. However, in vivo PDF only inhibited tumor fibrosis but not tumor growth and lung metastasis. A combination of PDF and doxorubicin, a chemotherapy drug, did inhibit tumor growth and lung metastasis [35]. In a previous study, the synergistic effect of doxorubicin and PDF was shown, suggesting a positive effect of the reduction of ECM components induced by PDF on doxorubicin blood perfusion and drug delivery [36].

The Hedgehog signaling pathway is involved in cell differentiation in embryonic cells. Reactivation of the Hedgehog pathway is observed in triple-negative breast cancer and promotes tumor growth and metastasis [37]. In the basal-like subtype of breast cancer, tumor cells stimulate the Hedgehog signaling pathway via paracrine signaling. Hedgehog-activated stromal cells further promote tumor growth and progression [38]. Hedgehog-ligand binds to its PTCH-receptor, which induces SMO-mediated translocation of Gli1 to the nucleus, leading to the transcription of target genes. Cazet et al. showed that the primary Hedgehog-activated stromal cells are CAFs, adding to the chemo-resistant stem cell-like phenotype of tumor cells. This phenotype is achieved via FGF5 expression and the production of fibrillar collagen. Inhibition of this pathway with SMO-inhibitors, vismodegib and sonidegib, made triple-negative breast cancer mouse model cells more sensitive to docetaxel [39]. Targeting of this specific interaction has been investigated in the EDALINE Clinical Trial, which will be discussed in the next section [40].

DNA damage induced by anti-cancer treatment might trigger stromal cells to enter senescence and thereby acquire the senescence-associated secretory phenotype (SASP). Production of amphiregulin (AREG) contributes to therapy resistance and leads to expression of programmed cell death 1 (PD-L1). PD-L1 expression is associated with immune checkpoint activation, creating an immunosuppressive tumor microenvironment. Both chemoresistance and immunosuppression decreased in prostate and breast cancer cells after targeting of AREG [41].

Another important cytokine produced by CAFs is SDF-1 or CXCL12. Binding of CXCL12 to its receptor CXCR4 on breast cancer cells is able to promote tumor cell proliferation and high levels of CXCL12 are associated with poor prognosis [42]. Various strategies antagonizing the effects of CXCR4 have been developed, but challenges like weak agonism or short half-life remain a problem [43]. The therapeutic effects of the novel antagonistic peptide E5 were recently evaluated [44]. This study showed that E5 was capable of inhibiting the interaction between 4T1 breast cancer cells and stromal cells mediated by CXCL12, leading to a reduction in migration and adhesion and enhancing the sensitivity of these cells to chemotherapeutics both in vitro and in vivo. Moreover, tumor microenvironment angiogenesis remarkedly decreased in a mouse breast cancer model. The pharmacokinetic stability of E5 was acceptable. However, the effect of E5 in human breast cancer models has not been investigated yet.

Tumor–stroma interactions are moreover important in metastases of breast cancer. The essential role of the fibrillar collagen receptor discoidin domain receptor 2 (DDR2) for the production of ECM and the organization of collagen fibers in CAFs has been shown in mice. DDR2 in tumor cells is also involved in the invasion process [45]. The small molecule inhibitor WRG-28 targets DDR2, thereby inhibiting tumor–stromal interactions as well as tumor invasion and migration. Thus, stromal targets also show potential in anti-metastasis treatment. To suppress both tumor progression and metastasis, combined treatment of chemotherapeutic and antimetastasis agents might have potential [46].

Because the possibilities of stromal targets in breast cancer treatment are just being discovered, the literature on this topic is limited and the development of these targets is in its infancy. Still little is known about differences in morphology and functionality of various stroma types. Better understanding of the complex effects and interactions of tumor–stroma and cancer cells is required for the translation of the stroma-targeting approach into established treatments. The findings described in this review might be developed and confirmed by further examining the efficacy and safety of the stromal-specific targets. Adequate confirmation of the potency of these strategies both in vivo and in vitro in human breast cancer models is an important prerequisite.

### Clinical needs

The effects of the tumor–stroma in different breast cancer subgroups might differ and needs to be determined. For further improvement of stromal-specific therapeutic strategies, different breast cancer subtypes should be taken into consideration. Special attention should be paid to stromal targets in triple-negative breast cancer, as the clinical need for therapy is high and high stromal content predicts poor prognosis in this aggressive subtype [13]. Three studies identifying promising stromal targets in TNBC are described in this review [30, 35, 37]. Both the improvement of these targets and the development of other targets in this subtype provide desired possibilities for future research.

## Clinical trials

Although the literature on stromal-specific targeting in breast cancer is limited, the amount of clinical trials that examine these strategies in patients is even more scarce. The EDALINE Clinical Trial is the only study that has been found in this review.

The EDALINE Study investigates targeting the Hedgehog pathway via combined SMO inhibition and docetaxel therapy in patients with advanced triple-negative breast cancer [40]. Sonidegib, a SMO-inhibitor, was approved for patients with metastatic or locally advanced basal cell carcinoma by the Food and Drug Administration of the United States and European Medicines Agency in 2015 [47]. 12 Patients with advanced triple-negative breast cancer were included in this study and assigned to accumulating doses. All patients previously received taxanes, the class of chemotherapeutics that includes docetaxel, as part of their therapeutic regimen. Anti-tumor effects were shown in three out of ten patients with measurable disease, all on the recommended phase II dose. One patient experienced complete response and two patients presented stabilized disease. However, the patient experiencing complete response was excluded from the trial based on docetaxel toxicity. These results do not give sufficient consideration to the effectiveness of this combination therapy, which is also due to the low number of included patients. Phase I clinical trials are not aimed at demonstrating therapeutic effectiveness and the efficacy of this combination therapy has yet to be shown. The safety of sonidegib and docetaxel combination therapy has been shown in this study, although four severe adverse events were observed in patients on the recommended phase II dose [40]. Future investigations are necessary to further validate the conclusions that can be drawn from this study, in additional phase I or conceivably in phase II or III studies.

## Possibilities for image-guided surgery and PET scanning

Besides stroma-specific cellular targeting, tumor–stroma also provides possibilities for the improvement of surgical treatment. Image-guided surgery is a novel strategy in cancer treatment encompassing real-time imaging of tumor cells during surgery, in addition to SPECT and PET which provide pre-operative imaging of tumor biology and location. This multidisciplinary technique provides new possibilities for enhancing cancer diagnostics and therapeutics. Fluorescent labels attached to tumor cell-binding molecules visualize the tumor cells to the surgeon. Both detection of microscopic tumors or residual lesions and determination of free resection margins are facilitated by this method [48]. The benefits of fluorescence image-guided surgery has already been shown in breast cancer and other cancer types [49]. As the importance of tumor–stroma in cancer development has been shown, fluorescent targeting of stromal cells for image-guided surgery has recently been a subject of interest. Among other cells, recent advances have been made in the identification of candidate targets on CAFs, including FAP and platelet-derived growth factor [50].

Furthermore, imaging of tumor–stroma could be extended using PET scanning. The field of theranostics explores the use of a highly specific agent for both therapeutic and diagnostic purposes, improving targeted and personalized therapy. Recent advances have been made in nanotheranostics for targeted treatment of triple negative breast cancer cells [51]. Targeting of CAFs in tumor–stroma might present a promising strategy, considering their active role in tumor development. Loktev et al. developed the small-molecule radiotracer FAPI-02, based on a high-affinity FAP inhibitor. Both in vitro and in vivo FAPI-02 showed high specificity and strong internalization into human and murine FAP-expressing cells. High tumor uptake of FAPI-02 was shown in xenograft-bearing mice and patients with metastasized breast, lung and pancreas carcinomas. Furthermore, the tracer did not accumulate in healthy tissues and was cleared from the body rapidly [52]. However, FAP is not only expressed in various cancer types, but also during wound healing, at arthritis inflammation sites and in atherosclerotic plaques [53]. This should be kept in mind when interpreting the results of imaging with radiolabeled FAPI-02.

## Conclusion

The contribution of tumor–stroma to breast cancer development, invasion and metastasis is increasingly recognized and understood. Both direct and indirect interactions between tumor and stromal cells have been shown to influence these processes. High stromal content in the primary tumor is described as prognostic parameter. Recent advances in the literature have suggested the importance of the tumor–stroma in resistance to chemotherapy. Cancer-associated fibroblasts are a major component of tumor–stroma and their role in cancer development and resistance is an emerging area in breast cancer research. Several stroma-specific therapies have been investigated, targeting both stromal cells and tumor–stroma interactions. However, stroma-specific therapy is warranted but still underdeveloped and merely one clinical trial evaluating this therapy type has been found. Tumor–stroma also provides possibilities for the improvement of breast cancer diagnosis and surgical treatment via fluorescent-label image-guided surgery and PET scanning.

The recent advances in stroma specific targets and the small insight into their possible clinical value discussed in this review provide an overview of the state-of-the-art of stromal targets in breast cancer therapy. Because this is a relatively new research area, the literature is limited. Therefore, stromal targets for breast cancer therapy provide possibilities for future research. Better understanding of tumor–stroma and tumor–stroma interactions is necessary for the development of agents that target these stromal molecules and interactions. Further research is needed to improve the strategies and agents discussed in this review. Moreover, conducting clinical trials examining the effects of the targets discussed in this review, like the EDALINE Trial, is a fundamental issue. The potential application of tumor–stroma in image-guided surgery and PET scanning should be explored. Taking into account tumor heterogeneity and breast cancer subtypes is important to generate new stroma-specific targeted therapies.
